# Deregulated miR-487b-3p in Patients with Non-Alcoholic Fatty Liver Disease and Its Regulatory Effect on Insulin Resistance

**DOI:** 10.5152/tjg.2026.25649

**Published:** 2026-03-16

**Authors:** Mingtao Chen, Jie Tan, Min Huang, Ting Zhan, Zheng Han, Meng Liu, Yuanyuan Lu, Xia Tian

**Affiliations:** Department of Gastroenterology, Wuhan Third Hospital,Tongren Hospital of Wuhan University, Wuhan, China

**Keywords:** Insulin receptor substrate 1, miR-487b-3p, nonalcoholic fatty liver disease

## Abstract

**Background/Aims:**

This study aims to elucidate both the diagnostic potential of miR-487b-3p for insulin resistance and its regulatory mechanisms in patients with nonalcoholic fatty liver disease (NAFLD).

**Materials and Methods:**

The study group included 60 NAFLD patients and 60 healthy controls. The expression levels of miR-487b-3p and insulin receptor substrate 1 (IRS1) in serum and cells were quantified by RT-qPCR. The diagnostic utility of the microRNA was evaluated through receiver operating characteristic (ROC) analysis. Glucose uptake in HepG2 was measured using fluorescence-based assays. The interaction between miR-487b-3p and IRS1 was confirmed by dual-luciferase reporter assay.

**Results:**

Compared with controls, NAFLD patients exhibited significantly elevated serum miR-487b-3p levels (*P *< .05). The ROC analysis demonstrated high diagnostic accuracy of miR-487b-3p in distinguishing between healthy controls and NAFLD patients. Functional analyses demonstrated that miR-487b-3p overexpression suppressed both glucose uptake and IRS1 expression (*P *< .05), whereas miR-487b-3p inhibition enhanced glucose uptake and IRS1 expression (*P *< .05). Mechanistically, miR-487b-3p negatively regulated IRS1 to modulate glucose uptake.

**Conclusion:**

miR-487b-3p may serve as a potential diagnostic biomarker for NAFLD, and miR-487b-3p negatively regulates IRS1 to modulate glucose uptake, thereby impairing insulin sensitivity in NAFLD patients.

**Main Points**

- Serum miR-487b-3p was significantly elevated in nonalcoholic fatty liver disease (NAFLD) patients.
- miR487b-3p is a potential diagnostic marker for NAFLD.
- Overexpression of miR-487b-3p inhibited glucose uptake and insulin receptor substrate 1 (IRS1) expression, while its inhibition promoted these effects.
- miR-487b-3p negatively regulates IRS1 to modulate glucose uptake.

## Introduction

Non-alcoholic fatty liver disease (NAFLD) constitutes a chronic hepatic disorder characterized by predominant triglyceride deposition within hepatocytes. This condition arises independently of steatogenic medications or hereditary disorders, progressing through a clinical continuum ranging from isolated steatosis to steatohepatitis with potential cirrhosis.^1^ Clinical manifestations include but are not limited to fatigue, anxiety, polydipsia, and abdominal distension.^2^ Current estimates indicate a global NAFLD prevalence of 25-30%, affecting over 1.8 billion people worldwide.^3^ Screening modalities encompass serum ALT, hepatic ultrasonography, liver biopsy, and MRI, however, all have limitations in specificity, sensitivity, and cost-effectiveness.^4^

NAFLD arises from synergistic genetic and environmental influences,^5^ with emerging evidence implicating epigenetic mechanisms in its pathogenesis. Recent studies highlight ethnicity-specific susceptibility, as demonstrated by Bonacini et al, reporting increased NAFLD risk among Hispanic populations.^6^ MicroRNAs (miRNAs), short non-coding RNAs of 19-28 nucleotides, epigenetically regulate gene expression central to NAFLD development.^7^ Substantial research confirmed that miRNAs modulate peripheral insulin action primarily through targeting key insulin signaling proteins, contributing to insulin resistance. Specific miRNAs exhibit diagnostic and mechanistic significance: circulating miR-122^8^ and miR-192 differentiate NAFLD patients from healthy controls, while miR-193a-5p modulates hepatic oxidative stress responses.^9^ MiR-125b-5p ameliorates hepatic steatosis by downregulating lipid metabolism factors via ESRRA suppression,^10^ and miR-690 improves insulin resistance by inhibiting NADK, thereby activating JAK/STAT and insulin signaling pathways to enhance AKT phosphorylation.^11^ Although primarily recognized as an oncogenic miRNA in gliomas,^12^ miR-487b-3p also influences skeletal myogenesis and myocyte differentiation. Insulin modulates glucose homeostasis through its effects on skeletal muscle, hepatic tissue, and white adipose depots, where insulin receptor substrate 1 (IRS1) mediates key signaling cascades.^13^

Quantitative PCR analysis revealed significant differential expression of miR-487b-3p between healthy controls and NAFLD patients. In this investigation, the mechanistic role of miR-487b-3p in the pathogenesis of insulin resistance was elucidated. The findings demonstrated that miR-487b-3p directly targets the 3′-untranslated region (3′-UTR) of IRS1 mRNA, consequently modulating cellular glucose uptake capacity. These results provide novel molecular insights into microRNA-mediated regulation of insulin resistance.

## Materials and Methods

### Patients and Clinical Data

This study enrolled 60 patients with confirmed NAFLD (32 males and 28 females, aged 30-54 years) from Wuhan Third Hospital, Tongren Hospital of Wuhan University Hospital, between October 2024 and April 2025, with the sample size determined through a priori power analysis using G*Power 3.1 (Heinrich-Heine; Dusseldorf, Germany) to achieve 95% statistical power (*α* = 0.05). The NAFLD was diagnosed based on abdominal ultrasound, which was defined as diffuse hyperechoic near-field liver parenchyma (brighter than the kidney and spleen regions), gradual attenuation of far-field echoes, and any of the following findings: unclear visualization of intraparenchymal structures; mild to moderate hepatomegaly with rounded borders; reduced blood flow signals but normal blood flow distribution; or incomplete or unclear visualization of the right hepatic lobe and diaphragm. Laboratory tests and liver biopsy (NAFLD Activity Score (NAS) ≥5; METAVIR score of fibrosis ≥F2) were also performed to confirm NAFLD diagnosis and eligibility for inclusion in the study. Consecutive sampling was employed to select participants. Exclusion criteria included: cirrhosis or portal hypertension, diabetes mellitus, autoimmune hepatitis, hepatocellular carcinoma, the presence of hepatitis B or C viral markers, alcohol consumption, or ischemic heart disease.

Additionally, 60 age- and sex-matched apparently healthy blood donors (30 males, 30 females) were recruited as controls. Participants in the control group had no serological evidence of viral hepatitis, had laboratory parameters within normal reference ranges, and exhibited no pathological abnormalities on diagnostic abdominal ultrasonography. This study was performed in line with the principles of the Declaration of Helsinki. Approval was granted by the Ethics Committee of Wuhan Third Hospital, Tongren Hospital of Wuhan University (Date. August 24, 2023; No.MS-2023-1014). In addition, for investigations involving human subjects, informed consent has been obtained from the participants involved.

### Blood Sampling

Following an overnight fast (≥8 hours), venous blood was collected using BD Vacutainer Red Top plain tubes (Dickinson and Company, USA). Samples were clotted at room temperature to separate serum, immediately aliquoted into cryogenic vials (Axygen®, USA), and stored at −70°C in ultra-low temperature freezers (Thermo Scientific, USA) until analysis.

Serum glucose, triglycerides (TG), high-density lipoprotein cholesterol (HDL-C), total cholesterol (TC), and low-density lipoprotein cholesterol (LDL-C) were quantified by the Model 7600 Series AUTOMATIC ANALYZER (Hitachi, Japan). The insulin levels were determined by electrochemiluminescence immunoassay on a Roche e-601 Analyzer (Roche Diagnostics Products Co., Ltd, Germany).

### Bioinformatics

Putative target genes of the miR-487b-3p were systematically identified through integrated analysis of the TargetScan (https://www.targetscan.org/vert_71/) and miRDB (https://www.mirdb.org/miRDB) datasets. The potential binding sites between miR-487b-3p and IRS1 were further predicted using TargetScan.

### RT-qPCR Assay

Total RNA was isolated from serum using the miRNeasy Serum/Plasma Kit (Qiagen, Germany) and from cells using TRIzol reagent (Takara, Japan), strictly adhering to the manufacturers’ protocols. The cDNA synthesis was performed using the PrimeScript RT Reagent Kit (Takara Bio, Japan), followed by quantitative PCR analysis with TB Green Premix Ex Taq II (Takara Bio, Japan) on a real-time PCR system. miR-487b-3p was detected using the PrimeScript RT kit. Reverse transcription was performed using its specific stem-loop RT primers, followed by RT-qPCR quantification using SYBR Green II Master Mix. U6 serves as an endogenous standard control.

### Receiver-Operating Characteristic Curve Analysis

To evaluate the diagnostic efficacy of miR-487b-3p for NAFLD, ROC curves were constructed based on miR-487b-3p expression levels using SPSS software.

### Cell Culture and Transfection

HepG2 cells (Procell, China) were cultured in Dulbecco’s Modified Eagle Medium (DMEM) (Procell, China) supplemented with 10% fetal bovine serum (FBS, Gibco, USA) and 1% penicillin-streptomycin (Beyotime, China), under standardized conditions (37°C, 5% CO₂).

HepG2 cell monolayers were established in 6-well culture plates (4 × 10⁵ cells/well), followed by overnight culture to achieve 70%-80% confluency. Cells were transfected with Lipofectamine 3000 transfection reagent (Fisher Scientific, USA) following the supplier’s technical specifications. DNA-lipid complexes were incubated with cells for 6 hours at 37°C/5% CO₂, after which the medium was replaced with fresh DMEM containing 10% FBS. Cells were maintained at 37°C/5% CO₂ for an additional 48 hours post transfection. Transfection efficiency and subsequent analyses were conducted at the 48-hour time point following transfection initiation.

### Glucose Uptake

HepG2 cells were seeded in 96-well plates at a density of 1 × 10⁴ cells/well. After 24 hours of culture (reaching ~80% confluency), cells were serum-starved for 12 hours in DMEM without serum. They were then treated with 100 nmol/L insulin for 24 hours. Glucose uptake was quantified using an assay kit (Cayman Chemical, Ann Arbor, MI) following the manufacturer’s protocol. Fluorescence was measured at 485 nm excitation/535 nm emission using a FlexStation 3 microplate reader (Molecular Devices, CA, USA).

### Dual-Luciferase Reporter Assay

Wild-type (WT) and mutant (MUT) sequences of the IRS1 3′UTR containing miR-487b-3p binding sites were cloned downstream of the firefly luciferase open reading frame to construct dual-luciferase reporter vectors. HepG2 cells were seeded in 24-well plates and co-transfected with the constructed vectors, along with either a miR negative control (miR NC), miR-487b-3p antagomir, or miR-487b-3p agomir, according to the manufacturer’s instructions. After 48 hours of transfection, dual-luciferase reporter assays were performed using a commercial kit (TransGen, China).

### Statistical Analysis

Statistical analysis was performed using SPSS 21.0 (IBM SPSS Corp.; Armonk, NY, USA). Data analysis was conducted using ROC curve analysis, and the diagnostic threshold was quantified through sensitivity/specificity determination. Data are expressed as mean ± standard deviation (SD). Student’s *t*-test or 1-way analysis of variance was used for comparisons between continuous variable groups, and the chi-square test was used for categorical data. Plotted using GraphPad Prism 10.1.2 () (GraphPad Software; Boston, Massachusetts, USA).

## Results

### Baseline Characteristics of the Participants

Table 1 presents the baseline demographic and clinical profiles of the enrolled cohort. Initial analysis revealed that NAFLD showed no significant association with age, marital status, gender, physical activity, smoking habits, or LDL-C levels (all *P *> .05). However, significant correlations were observed with body mass index (BMI), fasting insulin, fasting blood glucose (FBG), homeostasis model assessment of insulin resistance (HOMA-IR), TC, TG, and HDL-C levels (all *P *< .05). These findings indicated that metabolic parameters are critical determinants of NAFLD pathogenesis, warranting prioritized clinical intervention.

### Expression of MiR-487b-3p in Serum and Receiver-Operating Characteristic Curve Analysis

**Figure 1.**
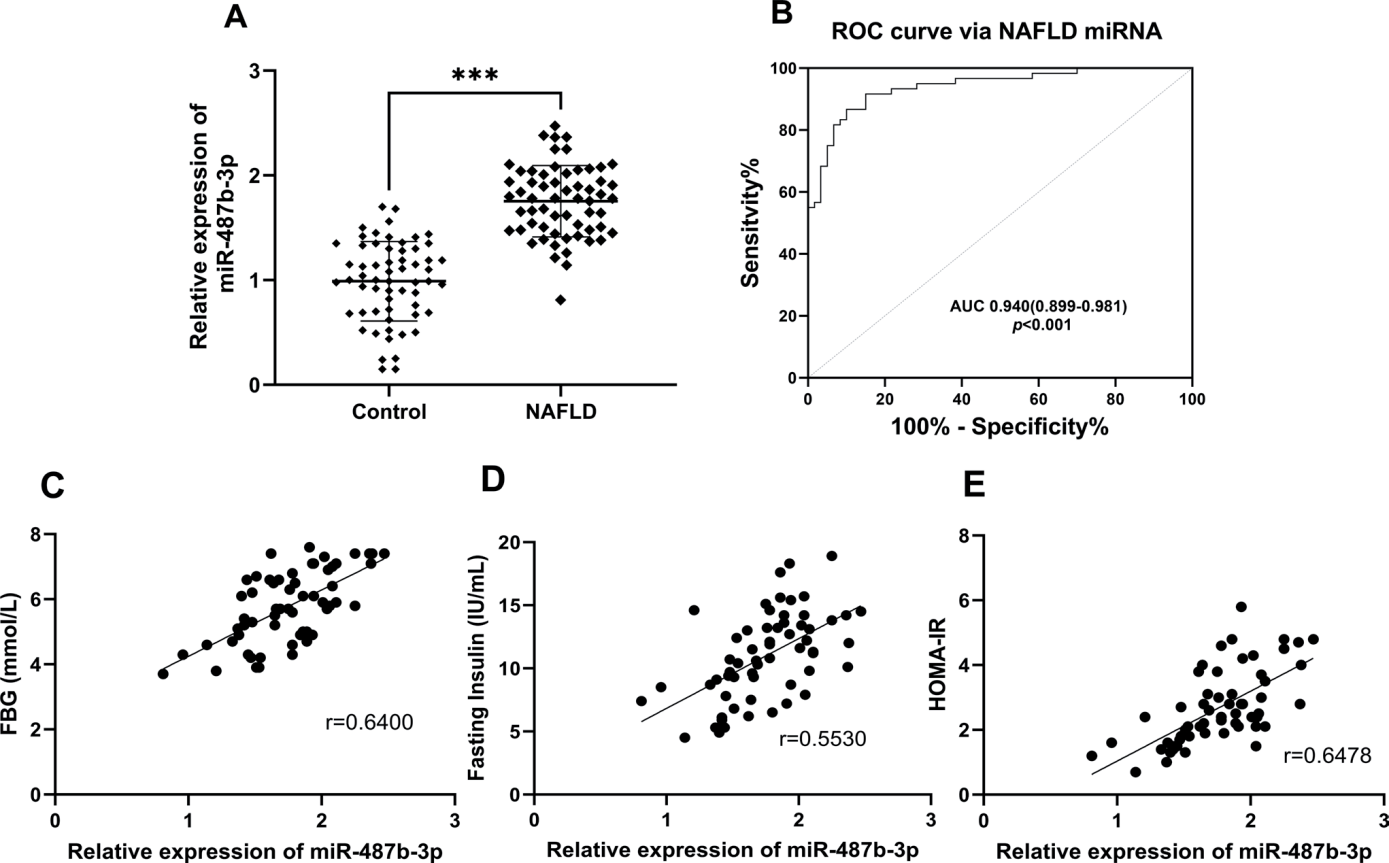
The miR-487b-3p expression in healthy controls and NAFLD groups. A. Relative expression of miR-487b-3p detected by RT-qPCR in healthy controls and NAFLD groups. B. ROC curve via miR-487b-3p levels. C. Correlation between miR-487b-3p and FBG. D. Correlation between miR-487b-3p and fasting insulin. E. Correlation between miR-487b-3p and HOMA-IR.

Analysis of serum miR-487b-3p expression revealed significantly elevated levels in patient groups compared with the healthy controls (*P *< .001) (Figure 1A). To assess the diagnostic potential of miR-487b-3p for NAFLD, ROC curve analysis was performed. The results revealed that miR-487b-3p exhibited an area under the curve (AUC) of 0.940 (95% CI, 0.899-0.981, *P *< .001), and the cutoff value was 1.365 (sensitivity, 91.67%; specificity, 85.00%) (Figure 1B; Supplementary Table 1). Biomarker’s stable discriminative performance was carried out through stratified 5-fold cross-validation (mean AUC = 0.933, SD = 0.048; Supplementary Table 2), demonstrating high diagnostic accuracy in discriminating NAFLD patients from healthy controls.

The correlation analysis results demonstrated that the expression levels of miR-487b-3p exhibited significant positive correlations with FBG (*r* = 0.6400, *P* < .001), fasting insulin (*r* = 0.5530, *P* < .001), and HOMA-IR (*r* = 0.6478, *P* < .001) (Figure 1C-E), suggesting that miR-487b-3p may play a regulatory role in glucose metabolism and insulin signaling pathways.

### MiR-487b-3p Modulates Glucose Uptake in Hepatocytes

**Figure 2.**
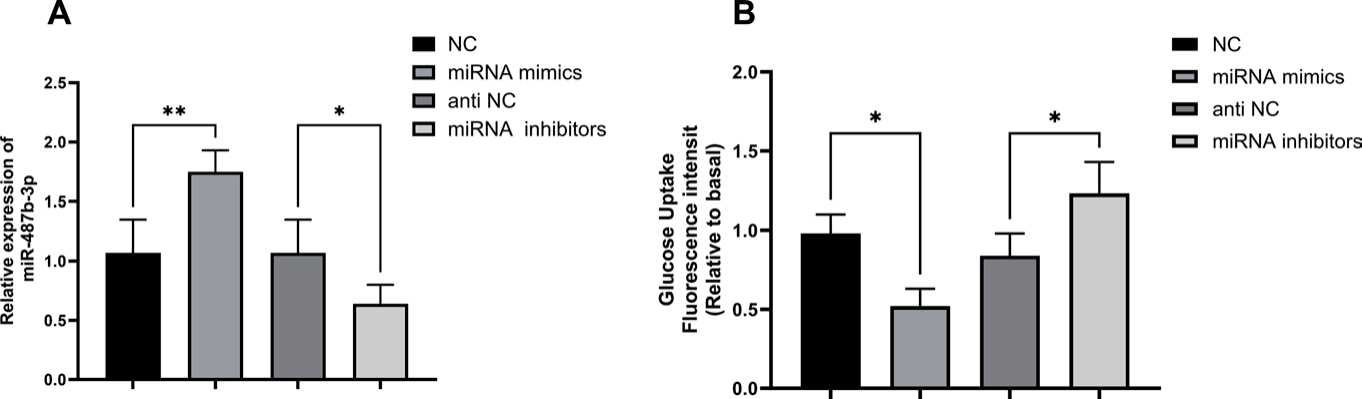
HepG2 cells were transfected with NC, anti-NC, miRNA mimics, and miRNA inhibitors for 48 h. A. Expression levels of miR-487b-3p in HepG2. B. Glucose uptake fluorescence intensity in HepG2.

Transfection of HepG2 cells with negative controls (NC), anti-NC, miR-487b-3p mimics, or miR-487b-3p inhibitor was performed to assess miR-487b-3p expression and glucose uptake rates, elucidating their functional relationship. Quantitative RT-PCR analysis demonstrated a statistically significant upregulation of miR-487b-3p expression in the mimics group compared to NC (*P* < .05), while the inhibitor group exhibited marked downregulation relative to anti-NC (*P *< .05) (Figure 2A). Fluorescence-based glucose uptake assays revealed that miR-487b-3p overexpression significantly impaired glucose absorption (*P* < .05), whereas its inhibition markedly enhanced uptake efficiency (*P *< .05) (Figure 2B), collectively establishing miR-487b-3p as a functional modulator of glucose metabolism in cells. These data provide compelling evidence for the regulatory role of miR-487b-3p in cellular glucose homeostasis through bidirectional modulation of uptake mechanisms.

### MiR-487b-3p Directly Targets the 3′-UTR of Insulin Receptor Substrate 1

Bioinformatic analysis using TargetScan and miRDB identified IRS1 as a potential target of miR-487b-3p.

**Figure 3.**
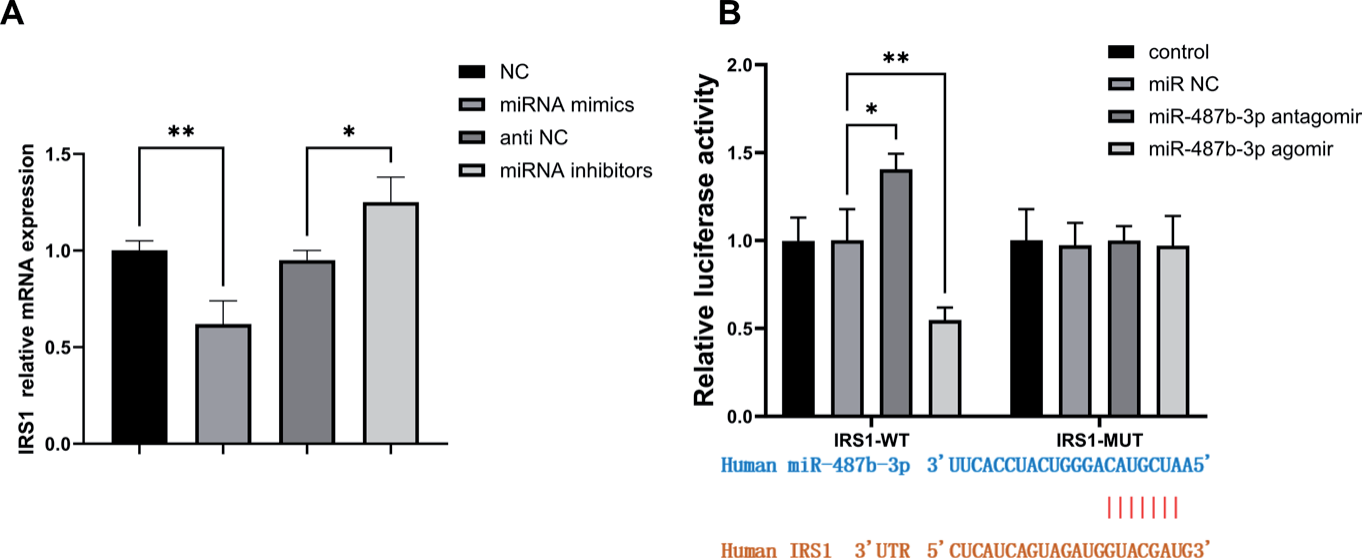
Targeting mechanism of miR-487b-3p on IRS1. A. Expression levels of IRS1 in HepG2. B. Correlation between miR-487b-3p and IRS1 expression in HepG2.

To investigate the regulatory relationship between miR-487b-3p and IRS1, HepG2 cells were transfected with NC, anti-NC, miR-487b-3p mimics, or miR-487b-3p inhibitor, followed by IRS1 mRNA quantification. The RT-PCR analysis revealed that miR-487b-3p overexpression significantly suppressed IRS1 expression compared to NC (*P *< .05), while its inhibition enhanced IRS1 levels versus anti-NC (*P *< .05) (Figure 3A). Subsequent dual-luciferase reporter assays in HepG2 cells, incorporating either wild-type or mutant IRS1 3′-UTR sequences, demonstrated that miR-487b-3p mimics reduced wild-type reporter activity by 42% versus miR-negative control (*P *< .01), whereas the inhibitor increased activity by 35% (*P *< .05). No significant changes occurred in mutant constructs across treatment groups (*P *> .05) (Figure 3B). These findings confirm direct targeting of IRS1 3′-UTR by miR-487b-3p, establishing its post-transcriptional regulatory mechanism through 3′-UTR binding.

## Discussion

Emerging as the predominant etiology of chronic liver disease (CLD),^14^ NAFLD exhibits a well-documented trajectory toward hepatocellular carcinoma (HCC) and hepatic cirrhosis. Accumulating evidence confirms significantly elevated HCC risk in NAFLD patients.^15^ Concurrently, these metabolic disturbances contribute to systemic metabolic dysregulation, including insulin resistance, enhanced gluconeogenesis, and increased blood glucose output. miRNAs are endogenous regulatory RNAs involved in cellular processes^16^ that modulate NAFLD pathogenesis through target mRNA interactions. For instance, studies have demonstrated that miR-122, a miRNA specifically highly expressed in the liver, whose knockdown effectively reduces excessive lipid production^17^ and alleviates inflamation;^18^ miR-192 can inhibit triglyceride synthesis in hepatocytes^19^ and significantly diminish lipid accumulation,^20^ while overexpression of miR-34a leads to triglyceride accumulation and impairs mitochondrial membrane potential.^21^ These mechanisms collectively influence NAFLD progression by directly modulating lipid synthesis and release, as well as inflammatory pathways, and can serve as biomarkers for NAFLD.^22^^-^^24^ While miR-122, miR-192, and miR-34a have been extensively studied in NAFLD, their focus has primarily been on lipid metabolism or inflammatory pathways, with limited direct involvement in the insulin signaling pathway. In this study, ROC analysis further confirmed that miR-487b-3p also has the potential to serve as a biomarker for NAFLD diagnosis. Additionally, it was found that miR-487b-3p was to some extent correlated with fasting insulin, FBG, and HOMA-IR levels, suggesting that miR-487b-3p may influence NAFLD by uniquely targeting the IRS1/PI3K/Akt axis through the insulin signaling pathway, and miR-487b-3p may play a pivotal role in regulating and ameliorating the dysregulated glucose-lipid metabolism and insulin resistance associated with NAFLD.

The pathogenesis of NAFLD primarily involves chronic nutrient oversupply and insulin resistance, which collectively promote intrahepatic fatty acid retention.^25^ Insulin resistance (IR) represents the core pathophysiological mechanism of NAFLD, and the pathophysiological foundation of IR stems from impaired hormonal signaling transduction within responsive cellular populations.^26^ Several studies indicated that attenuated stimulation of PI3K/Akt activity by insulin contributes to fasting hyperglycemia,^27^ characterized by reduced insulin signaling for hepatic glucose production suppression, affecting both the insulin receptor and downstream mediators,^28^ alongside diminished cellular glucose uptake efficiency, ultimately leading to hyperglycemia. An imbalance between energy intake and expenditure precipitates the emergence of IR;^29^ thus, insulin resistance precedes type 2 diabetes development , which is manifested by fasting hyperglycemia.^30^ Furthermore, hepatic de novo lipogenesis negatively correlates with systemic insulin sensitivity while and is associating with 24-hour plasma glucose and insulin concentrations.^31^ In this study, we demonstrated that miR-487b-3p modulates cellular glucose uptake, suggesting its potential role in influencing NAFLD through insulin signaling pathways associated with glucose metabolism.

Insulin receptor substrates (IRS) serve as pivotal molecular switches mediating hepatic insulin signaling and participate in orchestrating signal cascades.^32^ Among them, the IRS/PI3K/Akt signaling axis plays a critical role in glucose regulation. Upon insulin binding, IRS proteins are activated, subsequently recruiting and activating phosphatidylinositol 3-kinase (PI3K), which stimulates the downstream effector protein kinase B (Akt).^33^ The activated Akt directly influences the translocation of the glucose transporter, facilitating its movement from the cytoplasm to the plasma membrane to enhance glucose uptake, thereby modulating cellular glycogen storage, suppressing gluconeogenesis, and promoting lipid conversion.^34^ As a member of the IRS family, IRS1 mediates most metabolic actions of INSR activation. Its deficiency reduces glucose uptake in myocytes and impairs muscle glycogen synthesis, causing systemic glucose retention and consequently hepatic insulin resistance. Several studies showed that activation of the IRS1/PI3K/Akt pathway can alleviate insulin resistance in hepatocytes,^35^ and hepatic insulin responsiveness is closely associated with hepatic lipid accumulation and steatosis.^36^ Disruption of the IRS-1/PI3K/Akt pathway contributed to dysregulated lipid and glucose metabolism.^37^

In this study, we observed that miR-487b-3p can directly target the 3′ UTR of IRS1 mRNA, and that this interaction may potentially influence the IRS-1/PI3K/AKT signaling pathway, thereby possibly leading to impaired glucose uptake in hepatocytes. These findings suggested that circulating miR-487b-3p may amplify insulin resistance through a “liver-circulation” feedback mechanism, thereby perpetuating a metabolic vicious cycle. Consequently, targeting miR-487b-3p can ameliorate NAFLD pathology through multiple mechanisms, including the alleviation of IRS1 inhibition to activate the PI3K/Akt pathway, thereby enhancing cellular glucose uptake, improving fasting blood glucose levels, and restoring insulin sensitivity. It may mitigate or prevent lipid accumulation and inflammation arising from insulin signaling dysfunction. Whether miR-487b-3p exerts its role in NAFLD through the IRS1/PI3K/AKT signaling pathway remains a key focus of the future research.

In the current study, patients with type 2 diabetes mellitus (T2DM) were excluded, although IR is most closely associated with this subgroup. T2DM patients typically exhibit more severe IR and metabolic disturbances, accompanied by β-cell dysfunction and insufficient insulin secretion, leading to elevated fasting blood glucose levels. Given that these factors may confound the investigation of insulin signaling pathway mechanisms (e.g., glucose regulation), patients with T2DM were excluded during enrollment. However, they will be incorporated into future mechanistic validation experiments to systematically evaluate the synergistic effects of relevant miRNAs on the pathological progression of NAFLD in the context of IR combined with T2DM. Of course, validation in a larger independent cohort remains a necessary next step to provide supporting evidence.

In conclusion, the study demonstrated that miR-487b-3p negatively regulates IRS1 expression, thereby impairing glucose uptake in liver cells and contributing to insulin resistance pathways. Moreover, miR-487b-3p may serve as a diagnostic biomarker and a potential therapeutic target for NAFLD.

## Supplementary Materials

Supplementary Material

## Figures and Tables

**Table 1.** Baseline Characteristics of Patients with NAFLD

**Clinical Characteristic**	**Control Group (n = 60)**	**NAFLD Group (n = 60)**	**t/χ^2^**	***P***
Age	45.12 ± 5.86	42.70 ± 7.33	−1.865	.065
Gender			0.133	.715
Male	30(50.0%)	32(53.3%)		
Female	30(50.0%)	28(46.7%)		
BMI (kg/m^2^)	22.02 ± 2.15	29.29 ± 3.87	12.717	<.001
Smoking			0.048	.827
Yes	14	13		
No	46	47		
Marital status			0.240	.624
Yes	51	49		
No	9	11		
Exercise			0.436	.509
Yes	56	54		
No	4	6		
Fasting insulin (IU/mL)	9.11 ± 2.18	10.99 ± 3.46	3.558	.001
FBG (mmol/L)	5.21 ± 0.67	5.78 ± 1.10	3.442	.001
HOMA-IR	2.13 ± 0.56	2.66 ± 1.15	3.188	.002
TC (mmol/L)	4.15 ± 0.58	4.66 ± 0.73	4.173	<.001
TG (mmol/L)	1.10 ± 0.22	1.65 ± 0.69	5.873	<.001
HDL-C (mmol/L)	1.32 ± 0.22	1.18 ± 0.30	−4.173	<.001
LDL-C (mmol/L)	2.75 ± 0.37	2.73 ± 0.75	−0.129	.897

BMI, body mass index; FBG, fasting blood glucose; HDL-C, high-density lipoprotein cholesterol; HOMA-IR, homeostasis model assessment of insulin resistance; LDL-C, low-density lipoprotein cholesterol; NAFLD, nonalcoholic fatty liver disease; TC, total cholesterol; TG, triglycerides.

## Data Availability

The data that support the findings of this study are available on request from the corresponding author.
