# Supplementary material for: Deregulated miR-487b-3p in Patients with Non-Alcoholic Fatty Liver Disease and Its Regulatory Effect on Insulin Resistance
Source: Turk J Gastroenterol. 2026 Mar 16;37(5):629–36. doi: 10.5152/tjg.2026.25649 (PMC13182918; doi:10.5152/tjg.2026.25649)
Supplement: Supplementary Material [file supplementary_material.pdf]

Supplementary Table 1. ROC Curve Analysis of miR-487b-3p

Metric	Value
Sensitivity (%)	91.67
Specificity (%)	85.00
Cutoff value	1.365
AUC	0.940
95%CI	0.899-0.981
P value	<.001

Supplementary Table 2. Mean AUC Values of ROC Curves for the Serum Biomarker miR-487b-3p

	AUC1	AUC2	AUC3	AUC4	AUC5
AUC values	0.9375	0.9444	0.8542	0.9861	0.9444
Mean			0.9333		
Standard deviation			0.048		
